# Investigating genetic susceptibility to concussion through rare variants in ion channel and neurotransmission genes

**DOI:** 10.1007/s00415-026-14139-8

**Published:** 2026-09-12

**Authors:** Bridget H. Maher, Neven Maksemous, Heidi G. Sutherland, Robert A. Smith, Omar Dabash, Annette Greenhow, Fatima A. Nasralla, Dale R. Nyholt, Arn M. J. M. van den Maagdenberg, Rodney A. Lea, Lyn R. Griffiths

**Affiliations:** 1 Genomics Research Centre, Centre for Genomics and Personalised Health, School of Biomedical Sciences, Faculty of Health, Queensland University of Technology, Brisbane, QLD Australia; 2 Faculty of Law, Bond University, Gold Coast, QLD Australia; 3 Queensland Brain Institute, University of Queensland, St Lucia, QLD 4072 Australia; 4 Statistical and Genomic Epidemiology Laboratory, Centre for Genomics and Personalised Health, School of Biomedical Sciences, Faculty of Health, Queensland University of Technology, Brisbane, QLD Australia; 5 Department of Neurology, Leiden University Medical Center, Leiden, the Netherlands; 6 Department of Human Genetics, Leiden University Medical Center, Leiden, the Netherlands

**Keywords:** Concussion, Genetics, Ion channels, Neurotransmitters, Rare variants, Traumatic brain injury

## Abstract

Some individuals appear more susceptible to concussion or mild traumatic brain injury (mTBI) and the severity, range, and the persistence of post-concussion symptoms vary considerably between affected individuals. Genetic factors are likely to contribute to this variability. Symptomatic overlap of post-concussion syndrome with neurological conditions such as familial hemiplegic migraine (FHM) caused by rare pathogenic variants in ion channel and synapse protein genes, with high sensitivity to head trauma for some patients, suggests that variation in similar pathways may influence concussion susceptibility and recovery. To investigate this hypothesis, we performed whole exome sequencing in 93 unrelated individuals who had sustained a single or multiple concussions and examined rare protein-altering variants in FHM genes, other neuronal ion channel and transporter genes, and genes involved in neurotransmission. We identified 62 different rare missense variants across 24 genes in 59 participants (63%), with 26 individuals carrying 2 or more variants. The prevalence of specific likely damaging rare variants in the 16 ion channel-related genes that were identified was approximately fivefold higher than that observed from gnomAD population controls (Odds Ratio = 5.44, 95% CI [4.13,7.18], *P* < 0.0001). Notably, voltage-gated calcium and sodium channel genes, including *SCN9A*, together with neurotransmission-related genes such as *SNCAIP*, harboured multiple potentially deleterious variants. These findings suggest that rare deleterious variants in genes involved in ion homeostasis and neurotransmission may contribute to an individual’s susceptibility to concussion or more severe post-concussion symptoms. This study provides a foundation for future genetic and functional investigations aimed at improving our understanding of concussion susceptibility and outcomes. Further validation in larger cohorts and mechanistic studies is warranted to determine their utility as biomarkers of concussion risk and prognosis.

**Supplementary Information:**

The online version contains supplementary material available at https://doi.org/10.1007/s00415-026-14139-8.

## Introduction

Concussion is a condition of neurologic dysfunction resulting from biomechanical forces acting on the brain. Some individuals may be more susceptible to sustaining a concussion or display a range of neurological symptoms of varying severity. While most symptoms are transient and resolve in approximately 2 weeks, a subset of individuals develop post-concussion syndrome (PCS), experiencing symptoms such as headaches, dizziness, fatigue, and cognitive issues, that persist for months or even years [1–3]. Post-traumatic headache, often meeting the phenotypic criteria for migraine, is one of the most common symptoms following injury [4–6]. Why some people experience concussion with long-term neurological symptoms, and others do not, remains poorly understood. Inter-individual variability may be partly explained by injury severity, age, sex, or pre-injury health; however, there is growing evidence that genetic variation also influences both the likelihood of sustaining a concussion and the trajectory of recovery [7–9].

Previous small-scale genetic studies have investigated the association of concussion risk with genetic variants in genes with known neurological functions, including Alzheimer’s disease risk *APOE E4* allele [10–12] and *MAPT* [13, 14]; *BDNF* [15, 16]; glutamate transporters and receptors *SLC17A7* [17] and *GRIN2A* [18]; and the dopamine receptor gene *DRD4* [19]. Genome-wide association studies (GWAS) take a more agnostic approach, ideally with statistically robust sample sizes. In 2021, Kim et al. reported two gene loci with functions in neural development and axon outgrowth (*SPATA5* and *PLXNA4*) significantly associated with concussion risk [20]. GWAS by Kals et al. that used the extended Glasgow Outcome Scale at 6 months post-TBI as a measure of TBI outcome found 13 genetic loci associated at a suggested level of significance, with several biologically plausible associations such as *DOC2A*, which is involved in calcium-dependent neurotransmitter release, identified through supplementary transcription-wide association analysis [21]. Large GWAS of TBI in U.S. military veterans in 2024 identified 15 genome-wide significant loci mapping to 14 genes, including *APOE*, *NCAM1*, *FOXP2*, and *FTO*, and found that TBI showed high genetic correlation with psychiatric and risk-taking traits [22]. These studies, with a focus on common genetic variants, support the genetic underpinnings of various aspects of concussion, including the likelihood of sustaining TBI or concussion, recovery profile, and co-occurring symptoms.

Studies investigating the mechanisms of TBI indicate that substantial ion fluxes and alterations to neurotransmission occur following injury [23–25]. This is characterised by an efflux of potassium (K^+^), alongside the release of excitatory neurotransmitters such as glutamate, and an influx of sodium (Na^+^) and calcium (Ca^2+^) into neurons, resulting from altered membrane permeability following cellular distortion. These changes promote local and downstream neuronal depolarisation through the activation of voltage-gated ion channels and subsequent neurotransmission. Following these acute events, ATP-dependent ion pumps are activated to restore ionic homeostasis. This process substantially increases neuronal energy demand and oxidative stress [23]. The resulting metabolic strain can lead to hyperglycolysis, which is further exacerbated by Ca^2+^-mediated disruption of mitochondrial function. Neuronal energy depletion can last for approximately 2 weeks [26], aligning with the typical recovery period for concussion and highlighting the importance of these early molecular events in its pathophysiology. Changes in the subunit composition of ligand-gated ion channels following a TBI have also been reported in animal models [27–29] and may correlate with the altered cognition and learning displayed by concussion sufferers [30, 31]. Thus, neurotransmitters and ion channels are involved in the earliest stages of cellular response to TBI [32], and may represent key players in concurring severity after TBI at the cellular and metabolic level, and development of persistent symptoms including post-traumatic headache and migraine.

Further evidence of the potential role of genetic variation in concussion and its aftermath arises from the pathophysiological and symptomatic overlap of PCS with other neurological disorders, particularly the ion channelopathies. Familial hemiplegic migraine (FHM) is a rare autosomal dominant disorder in which migraine attacks are accompanied by motor dysfunction caused by rare pathogenic variants in ion channel, transporter, and synaptic genes [33]. Some individuals with FHM may have severe responses to trivial head trauma. For example, patients with FHM Type 1 (FHM1) caused by pathogenic variants in the calcium channel gene *CACNA1A* may exhibit high sensitivity to head impacts; in cases involving the severe p.Ser218Leu mutation, even minor head injury can result in concussion, seizures, cerebral oedema, coma, or death [34, 35]. This phenotype closely resembles second-impact syndrome, where a subsequent mild injury following an initial concussion can lead to catastrophic cerebral oedema. FHM1 *CACNA1A* mutations lead to a gain of Ca_V_2.1 channel function with increased Ca^2+^ neuronal influx [36]. Knock-in mouse models carrying human *CACNA1A* mutations display enhanced glutamatergic neurotransmission and an imbalance between excitatory and inhibitory signalling, which facilitates cortical spreading depression (CSD) and worsens outcomes after experimental TBI [37, 38]. Importantly, the dysregulated ion fluxes and neurotransmission that occur in FHM mirror the metabolic cascade observed post-TBI and may underlie acute post-concussive symptoms.

We therefore hypothesise that deleterious variants in genes involved in ion homeostasis and synaptic neurotransmission increase susceptibility to concussion and/or impair recovery following TBI, as these pathways are central to the neurometabolic response to concussion. To investigate this hypothesis, we performed whole exome sequencing (WES) in individuals who had sustained one or more concussions and examined rare predicted deleterious variants in genes encoding neuronal ion channels and transporters, and genes involved in neurotransmission. Focusing on pathways with well-established mechanistic roles in concussion pathophysiology, we identified rare and potentially damaging variants, including some with previously demonstrated functional effects. These findings suggest that genetic vulnerabilities within key neurometabolic pathways may contribute to concussion risk and associated sequelae, warranting further investigation in larger cohorts and functional studies.

## Materials and methods

### Ethics

The study was conducted in accordance with the Declaration of Helsinki; the protocol was approved by the Human Research Ethics Committee (HREC) of Queensland University of Technology (QUT) (Project approval number 1400000748), with informed consent obtained from all the participants. All experiments were performed according to the guidelines and regulation of QUT HREC.

### Cohort

A cohort of 93 unrelated individuals who had suffered one or more concussions was recruited to the study. Index cases were collected through four avenues:

1. *n* = 15 cases who had developed severe reactions to trivial head trauma and referred for FHM gene testing, but negative for pathogenic variants in known genes. Their clinical presentations and DNA collection have been described previously [39]. Sample identification (ID) numbers: R#.
2. *n* = 11 participants were recruited through Brisbane hospitals with diagnosed concussion. Sample IDs: BBB#.
3. *n* = 32 participants were recruited through local sporting groups and campus recruitment. Of these, 21 had concussion diagnosed by a medical professional and the remainder were self-reported. Twenty reported multiple concussions in the collected questionnaire data. Sample IDs: C#, CN#.
4. *n* = 35 Australian Defence Force veterans who had previously sustained concussion/s were recruited through the Gallipoli Medical Research Foundation—Greenslopes Private Hospital (GMRF-GPH) Biobank. Sample IDs: P#.

Response to concussion in the participants varied from no severe or long-lasting symptoms to persistent and ongoing symptoms including migraine, memory problems, fatigue, and vertigo from 3 months to > 1 year post-concussion event. Available information related to participants’ concussion history and symptoms is provided in Table S1. For some cases, family members were also recruited (*n*=15). Despite differences in recruitment source, all participants met the inclusion criterion of having sustained one or more concussions, allowing investigation of genetic factors that may operate across various concussion contexts rather than within a single exposure group.

### DNA extraction and whole exome sequencing

Genomic DNA was isolated from either whole blood samples using QIAamp DNA Blood Mini Kits (Qiagen, Hilden, Germany), or from saliva using ORAgene-DNA OG-500 kits and PrepIT reagent (DNA Genotek, Stittsville, Canada) as per the manufacturer’s instructions. We have previously shown that results for WES using DNA obtained from either blood or saliva are comparable [40]. Extracted blood DNA samples were available from the GMRF-GPH Biobank samples.

Next-generation sequencing libraries for WES were constructed using Ion AmpliSeqTM Exome RDY Library Kits (Cat. no. 4489837 Rev A.0, Thermo Fisher, Waltham, MA, USA) according to the manufacturer’s protocol. The libraries were sequenced on the Ion S5 plus sequencer, using Ion v550 Chips (Thermo Fisher Scientific). Sequence reads were aligned to the human reference genome (hg19), the variants were called and the VCF files were generated using the Ion Torrent Suite software (Thermo Fisher Scientific). The VCF files were then uploaded to the locally hosted Ion Reporter Server software (v5.12) for automated variant annotation and filtering. The Binary Alignment/Map (BAM) format files were uploaded into the Integrative Genomics Viewer (IGV v2.3) software (http://www.broadinstitute.org/igv), to visually examine potential variants.

### Ion channel and neurotransmitter gene selection and variant curation

Variant prioritisation from the WES data was performed using a sequential filtering strategy to identify rare coding variants in genes with biological relevance to concussion. Analysis was restricted to exome variants in three predefined gene sets (Tiers 1–3): (1) causally implicated in severe types of migraine *(CACNA1A, ATP1A2,* and *SCN1A)*; (2) ion channel and transporter genes (*n* = 353 genes); and (3) genes affecting neurotransmission (*n* = 220 genes). Gene lists for ion channel and neurotransmitter-related genes are shown in Table S2. The gene sets for each tier of analysis were applied by filter using Ion Reporter™ v5.12 software (Thermo Fisher Scientific). Tier 1 comprised the main FHM genes. For Tier 2, a filter chain was created of all genes with Gene Ontology (GO) [2017/11/01 release] molecular functions that included calcium channel, sodium channel, potassium channel, chloride channel, as well as ATPases related to ATP ion pump functions. For Tier 3, the term “neurotransmitter” was applied to capture all related GO terms including calcium ion-regulated exocytosis of neurotransmitter, neurotransmitter receptor transport, neurotransmitter secretion, neurotransmitter transport, neurotransmitter transporter activity, neurotransmitter biosynthetic process, etc. (Table S2).

Variants in these genes were then (i) limited to protein-altering coding variants; (ii) filtered for rarity, i.e. had minor allele frequency (MAF) <0.02 in both gnomAD c2.1.1 (exomes) as well as the non-Finnish European (NFE) genetic ancestry group; (iii) were required to be predicted deleterious by at least two in silico prediction tools (SIFT version 5.2.2 [41], PolyPhen version 2.2.2 release 405c [42], MutationTaster2021 [43], and CADD score based upon Ensembl Ch37 [44]); and (iv) passed visual confirmation in IGV (v2.5.3). Finally, following automated filtering, variants were prioritised if they occurred in genes with variants identified in two or more unrelated participants, followed by manual review in the context of published literature and known gene function to assist with biological relevance and interpretation.

### Genotyping of selected variants in a migraine case–control cohort

To assess frequencies of the channel-related variants detected in the concussion cohort in independent samples some genes/variants of interest in *ATP7B*, *CACNB2*, *SCN8A*, *SCN9A*, and *SCN10A* were prioritised for genotyping in an Australian cohort of 280 migraine cases and 280 sex- and age-matched controls. Genotyping was performed via a multiplex MassARRAY assay (Agena Bioscience, San Diego, CA, USA). Primers were designed using Assay Design Suite v3.0 software for: *ATP7B* variants c.122A>G (p.Asn41Ser), c.1555G>A (p.Val519Met), c.2383C>T (p.Leu795Phe), c.2972C>T (p.Thr991Met), c.3700G>T (p.Val1234Phe), and c.3859G>A (p.Gly1287Ser); *CACNB2* variants c.503G>A (p.Cys168Tyr) and c.1357C>T (p.Leu453Phe); *SCN8A* variant c.3076C>T (p.Arg1026Cys), *SCN9A* variants c.684C>G (p.Ile228Met), c.1109C>T (p.Thr370Met), c.3734A>G (p.Asn1256Ser) and c.3799C>G (p.Leu1278Val); and *SCN10A* variants c.2530C>T (p.Arg844Cys), c.3859G>A (p.Val1287Ile), and c.4585G>A (p.Ala1529Thr) with sequences available upon request. Not all variants of interest were genotyped due to assay designability or cost constraints. Primers were obtained from Integrated DNA Technologies (Singapore). Genotyping was conducted according to manufacturer’s protocols using PCR and iPLEX MassARRAY reagents on a matrix-assisted laser desorption ionization time-of-flight (MALDI-TOF) mass spectrometry platform (Agena Biosciences, San Diego, CA, USA). Genotypes were called with Typer 4.0 software. Assays for *SCN9A* p.Ile228Met and *SCN10A* p.Val1287Ile variants failed and were not further analysed.

### Rare variant burden analysis

Rare variant burden analysis focused on the rare variants identified in the known FHM genes and other ion channel genes identified in this study. Counts of these variants were compared with the numbers observed in a general population control group obtained from the gnomAD database, selecting non-Finnish Europeans as most similar to the ancestral background of the recruitment population in Australia, and using a Fisher’s exact test to determine statistical significance. Odds ratios were calculated as an estimate of effect size.

## Results

### Single-variant analysis

Annotated WES data from 93 unrelated individuals who have sustained one or more concussions were filtered to focus on rare (MAF <0.02), protein coding variants in known causative migraine genes, ion channel genes, or genes affecting neurotransmission (Table S2) in accordance with our hypothesis that genetic vulnerabilities in these genes contribute to increased concussion risk. A variant filtering workflow showing the number of variants retained after each filtering step is shown in Fig. S1. Rare amino acid changing variants that were predicted to be damaging according to at least two of the in silico prediction tools used (SIFT, Polyphen, MutationTaster, and CADD) were identified. 61.5% of these were predicted to be damaging by all four tools. In total, 62 rare missense variants were identified across 24 genes in 59 (63%) participants, with 26 participants carrying 2 or more candidate variants. All identified variants were heterozygous in participants. Tables 1, 2 and 3 detail the 62 variants identified in the three tiers of analysis: (1) known migraine causative genes; (2) ion channel and transporter genes; and (3) genes affecting the neurotransmission, respectively, and include results of in silico predictions, summaries of current ClinVar classifications, and the gnomAD MAF for each in non-Finnish Europeans. Genes carrying identified variants included calcium channels (*CACNA1A, CACNA1B, CACNA1C, CACNA1D, CACNA1E, CACNA1I, CACNB2*), sodium channels (*SCN1A, SCN8A, SCN9A, SCN10A*), ATPases (*ATP10A, ATP13A4, ATP7B*), potassium channels (*KCNJ10, KCNT2*) and various genes involved in neurotransmission (*CHAT, DAGLB, KIF17, NRG1, SNCAIP, SV2C, SYT9, UNC13B*).

**Table 1:** Details of rare genetic variants identified in known migraine causative genes

Gene	Variant details							Bioinformatic predictions				GnomAD NFE data		
	Locus	Ref	Amino Acid Change	Coding change	SNP ID	CLINVAR	Samples with Variant	SIFT pred	Polyphen2 HDIV pred	Mutation Taster pred	CADD phred	Variant Allele count	Total Allele count	Freq
*CACNA1A*	19:13,428,124	C	p.Ala454Thr	c.1360G>A	rs41276886	likely benign (14) – Condition: Various neurological disorders	CN7 P034 P038	T	D	D	24.1	265	47,808	0.005543
*SCN1A*	2:166,848,003	G	p.Arg1917Gly	c.5749C>G	rs121917956	likely benign (12) – Condition: Various neurological disorders	CN11 C2	D	D	D	22.0	67	48,282	0.001388

SIFT is based on version 5.2.2; PolyPhen is based on version 2.2.2 release 405c; MutationTaster2021; CADD score is based upon Ensembl Ch37

*GnomAD NFE* allele frequency of non-Finnish Europeans, *GnomAD* allele count is based upon v2.1.1 (exomes)

**Table 2:** Details of rare genetic variants identified in ion channel and ion transporter genes

Gene	Variant details							Bioinformatic predictions				GnomAD NFE data		
	locus	Ref	Amino acid change	Coding change	SNP ID	CLINVAR	Samples with variant	SIFT pred	Polyphen2 HDIV pred	Mutation taster pred	CADD phred	Variant allele count	Total allele count	Freq
*ATP10A*	15:25,947,181	G	p.Ala881Val	c.2642C>T	rs142704035		R170^a^	D	D	D	32	16	113,700	0.000141
	15:26,108,002	T	p.Glu81Gly	c.242A>G	rs368768805		CN27	D	D	D	24.7	11	107,790	0.000102
*ATP13A4*	3:193,132,450	T	p.Asn978His	c.2932A>C	rs770185624		CN18	T	B	D	22.3	5	113,740	0.000044
*ATP7B*	13:52,511,656	C	p.Gly1287Ser	c.3859G>A	rs762866453	Conflicting classifications of pathogenicity (6) relating to Wilson Disease	P254	D	D	D	27.6	1	113,286	0.000009
	13:52,511,815	C	p.Val1234Phe	c.3700G>T	rs193922108		P018	D	D	D	33	0	113,194	0.000000
	13:52,520,508	G	p.Thr991Met	c.2972C>T	rs41292782	Conflicting classifications of pathogenicity (9) relating to Wilson Disease	C4	D	D	D	33	266	112,746	0.002359
	13:52,531,716	G	p.Leu795Phe	c.2383C>T	rs751710854	Pathogenic/Likely pathogenic (9) relating to Wilson Disease	R211^b^	D	D	D	28.3	4	113,288	0.000035
	13:52,542,732	C	p.Val519Met	c.1555G>A	rs192957846	Uncertain Significance (5) relating to Wilson disease	CN25	D	D	D	29.3	127	113,118	0.001123
	13:52,549,234	T	p.Asn41Ser	c.122A>G	rs201738967	Pathogenic/Likely pathogenic (12) relating to Wilson Disease	P224	D	D	D	24.4	55	113,168	0.000486
*CACNA1B*	9:140,952,560	G	p.Arg1389His	c.4166G>A	rs184841813	Uncertain Significance (2)	BBB19	D	P	D	34	78	113,040	0.000690
	9:141,015,296	C	p.Ser2151Leu	c.6452C>T	rs200034261	Uncertain Significance (2)	P235	D	D	D	29.9	37	75,310	0.000491
*CACNA1C*	12:2,690,844	A	p.Ile662Leu	c.1984A>C			R259^c^	D	B	D	23.1			
	12:2,774,859	A	p.Tyr1552Cys	c.4655A>G	rs2153709630	Uncertain Significance (1) – Condition: Long QT Syndrome	C5	D	D	D	32.0			
	12:2,800,220	A	p.Asn2174Ser	c.6521A>G	rs201090446	Conflicting classifications of pathogenicity (9)	BBB12	D	D	D	24.2	69	104,020	0.000663
*CACNA1D*	3:53,787,624	A	p.Asn1254Ser	c.3761A>G	rs147146258		P186	T	B	D	22.9	78	113,768	0.000686
	3:53,837,487	C	p.Arg1845Trp	c.5533C>T	rs144688228	Conflicting classifications of pathogenicity (3) – Condition: not Provided	CN4	D	B	D	23.6	17	113,770	0.000149
	3:53,844,173	G	p.Gly2034Ser	c.6100G>A	rs368340190	Uncertain Significance (1) – Condition: not Provided	CN15	T	B	D	20.5	7	113,558	0.000062
*CACNA1E*	1:181,706,777	C	p.Ser1180Leu	c.3539C>T	rs202226007		P200	D	D	D	34	4	112,714	0.000035
	1:181,759,588	C	p.Arg1932Trp	c.5794C>T	rs776416674		BBB12	D	D	D	26.8	6	112,858	0.000053
	1:181,767,528	G	p.Arg2167Gln	c.6500G>A	rs370385055	Conflicting classifications of pathogenicity (2) – Condition: not Provided	P242	D	P	D	34	7	112,966	0.000062
*CACNA1I*	22:40,045,653	C	p.Ser537Leu	c.1610C>T	rs372904212		P191	T	D	D	22.5	6	44,670	0.000134
	22:40,081,856	G	p.Asp2005Tyr	c.6013G>T	rs771105041		P186	D	D	N	29.4	42	27,346	0.001536
	22:39,994,250	C	p.Arg111Gly	c.331C>G	rs751729397		R211^b^	D	P	D	34	1	103,132	0.000010
*CACNB2*	10:18,789,787	G	p.Cys168Tyr	c.503G>A	rs773215003	Uncertain Significance (4) – Condition: various cardiovascular conditions	P200	D	D	D	26.8	2	113,632	0.000018
	10:18,827,118	G	p.Asp438Asn	c.1312G>A	rs778466195	Uncertain Significance (2) – Condition: cardiovascular phenotype and Brugada syndrome 4	C2	D	D	D	34	1	113,742	0.000009
	10:18,827,163	C	p.Leu453Phe	c.1357C>T	rs145638628	Conflicting classifications of pathogenicity (4) – Condition: cardiovascular phenotype and Brugada syndrome 4	CN29	D	D	D	29.0	48	113,762	0.000422
*KCNJ10*	1:160,011,262	T	p.Lys354Arg	c.1061A>G	rs142596580	Uncertain Significance (9) – Condition: Condition: various including EAST syndrome and non-syndromic hearing loss	CN32	D	D	D	26.7	54	113,678	0.000475
	1:160,012,271	G	p.Arg18Trp	c.52C>T	rs138457635	Uncertain Significance (7) – Condition: Condition: various including EAST syndrome and non-syndromic hearing loss	R117^d^	D	D	D	27.7	78	113,716	0.000686
*KCNT2*	1:196,250,115	T	p.Lys929Glu	c.2785A>G	rs1659036452		CN7	D	P	D	22.6			
	1:196,309,627	T	p.Ile543Leu	c.1627A>C	rs780602272		P095	T	B	D	23.8	5	113,102	0.000044
*SCN9A*	2:167,089,942	G	p.Leu1278Val (p.Leu1267Val)*	c.3832C>G	rs180922748	Conflicting classifications of pathogenicity (16) – Condition: Various including pain and seizure disorders	CN10	D	D	D	26.5	212	102,260	0.002073
	2:167,094,638	T	p.Asn1256Ser (p.Asn1245Ser)*	c.3767A>G	rs141268327	Conflicting classifications of pathogenicity (24) – Condition: Various including pain and seizure disorders	R211^b^ P026R171^e^	D	D	D	23.9	837	111,518	0.007506
	2:167,108,386	G	p.Arg1121Trp (p.Arg1110Trp)*	c.3361C>T	rs190664764	Conflicting classifications of pathogenicity (6) – Condition: pain and seizure disorders	R256	D		N	24.0	166	112,256	0.001479
	2:167,137,020	C	p.Trp730Cys (p.Trp719Cys)*	c.2190G>C	rs202055175	Conflicting classifications of pathogenicity (15) – Condition: pain and seizure disorders	BBB7	D	D	D	34	3	91,662	0.000033
	2:167,145,152	G	p.Thr370Met	c.1109C>T	rs200391162	Conflicting classifications of pathogenicity (5) – Condition: pain and seizure disorders	BBB27	D	D	D	32	1	102,700	0.000010
	2:167,160,752	G	p.Ile228Met	c.684C>G	rs71428908	Conflicting classifications of pathogenicity (16) – Condition: pain and seizure disorders	CN10	D	D	D	22.8	194	113,030	0.001716
*SCN10A*	3:38,743,402	C	p.Ala1529Thr	c.4585G>A	rs757916036		P232	D	D	D	34	1	113,512	0.000009
	3:38,753,882	C	p.Val1287Ile	c.3859G>A	rs145032037		CN1	D	D	D	34	126	113,524	0.001110
	3:38,755,450	C	p.Arg1268Gln	c.3803G>A	rs138832868	Conflicting classifications of pathogenicity (7) – Conditions: pain disorders and cardiovascular phenotypes	BBB3	D	B	D	25.5	313	113,532	0.002757
	3:38,770,143	G	p.Arg844Cys	c.2530C>T	rs140158387	Uncertain significance (2)—Conditions: Cardiovascular phenotypes	P307	D	D	D	35	2	113,548	0.000018
*SCN8A*^f^	12:52,162,823	C	p.Arg1026Cys	c.3076C>T	rs117217073	Conflicting classifications of pathogenicity (6) – Condition: seizure disorders	CN18 R167 BBB15 P194	D	D	D	24.5	1663	112,888	0.014730

SIFT is based on version 5.2.2; PolyPhen is based on version 2.2.2 release 405c; MutationTaster2021; CADD score is based upon Ensembl Ch37

*Variant has previously been described in the literature with alternate protein residue number as annotated against a different transcript

^a^R170 A rare variant in *ATP10A* was previously reported in Ibrahim et al. (2020) [39]

^b^R211 Rare variants in *ATP7B* and *CACNA1I* were previously reported in Ibrahim et al. (2020)

^c^R259 Rare variant in *CACNA1C* was previously reported in Ibrahim et al. (2020)

^d^R117 Rare variant in *KCNJ10* was previously reported in Ibrahim et al. (2020)

^e^R171 also carries a rare variant in *GRIK1* that is predicted to be deleterious as previously reported in Ibrahim et al. (2020), not listed here as no other sample in this cohort carries a rare, potentially deleterious variant in this gene

^f^Some regions of the *SCN8A* gene have poor WES coverage with Ion AmpliSeqTM Exome Kits, so some variants in this gene may be missed

*GnomAD NFE* allele frequency of non-Finnish Europeans, *GnomAD* allele count is based upon v2.1.1 (exomes)

**Table 3:** Details of genetic variants identified in neurotransmission-related genes

Gene	Variant details							Bioinformatic predictions				GnomAD NFE data		
	Locus	Ref	Amino acid change	Coding change	SNP ID	CLINVAR	Samples with variant	SIFT pred	Polyphen2 HDIV pred	Mutation taster pred	CADD phred	Variant allele count	Total allele count	Freq
*CHAT*	10:50,835,688	G	p.Arg323His	c.968G>A	rs200176236	Likely Benign (1) – Condition: familial infantile mysathenia	CN41	D	D	D	31	29	113,718	0.000255
	10:50,856,643	C	p.Leu458Phe	c.1372C>T	rs76014951	Benign/Likely Benign (6) – Condition: familial infantile mysathenia	CN32 P191	T	D	D	26.9	569	112,684	0.005050
	10:50,863,188	G	p.Arg561Gln	c.1682G>A	rs80097077	Benign/Likely Benign (7) – Condition: familial infantile mysathenia	CN11 BBB3 R118^a^	D	D	D	27.2	1188	113,628	0.010460
*DAGLB*	7:6,449,794	A	p.Ile596Thr	c.1787 T>C	rs139753251	Benign (1) – Condition: Not provided	CN26 P242	D	D	D	25.4	896	94,702	0.009461
*KIF17*	1:21,009,189	T	p.Glu807Ala	c.2420A>C	rs115257742		CN32	D	D	D	28.6	188	113,682	0.001654
	1:21,042,033	T	p.Arg111Gly	c.331A>G	rs35835983		CN6 BBB17	D	P	D	23	1966	113,480	0.017320
	1:21,044,007	C	p.Glu65Lys	c.193G>A	rs147438943		CN27	T	P	D	24.9	709	110,288	0.006429
*NRG1*	8:32,617,888	C	p.Thr416Ser	c.1247C>G	rs62497784	Likely Benign (1) – Condition: Not provided	BBB17	T	D	D	22.3	405	113,256	0.003576
*SNCAIP*	5:121,759,069	G	p.Val213Met	c.637G>A	rs201152226		P095	D	P	D	17.65	8	113,352	0.000071
	5:121,786,403	C	p.Arg621Cys	c.1861C>T	rs28937592	Likely Benign (4) – Condition: Parkinson disease	P160	D	D	D	27.5	620	113,618	0.005457
	5:121,786,667	G	p.Glu709Gln	c.2125G>C	rs55712196	Likely Benign (3) – Condition: Parkinson disease	CN28 C4 R222 P194 P178	D	D	D	24.6	1418	113,392	0.012510
	5:121,786,959	G	p.Arg806His	c.2417G>A	rs140850272	Benign (2) – Condition: Parkinson disease	R259 P027 P202	D	D	D	28.5	1552	113,672	0.013650
*SV2C*	5:75,581,035	T	p.Val321Glu	c.962 T>A	rs762537711		P186	D	P	D	29.2	3	113,238	0.000026
	5:75,591,631	G	p.Asp456Asn	c.1366G>A	rs756018103		C7	T	P	D	25.7	3	112,560	0.000027
*SYT9*	11:7,324,378	G	p.Arg85Gln	c.254G>A	rs773842068		R211	D	P	D	24.1	1	113,724	0.000009
	11:7,437,285	C	p.Leu353Val	c.1057C>G	rs117876446		CN1 P202 P200	D	D	D	25	1111	113,672	0.009774
*UNC13B*	9:35,380,598	G	p.Arg697Gln	c.2090G>A	rs144007866		P194	T	D	D	31	29	113,746	0.000255
	9:35,381,587	G	p.Ser760Ile	c.2279G>T	rs911067546		R150^b^	D	P	D	20.9			
	9:35,396,516	C	p.Asn1035Lys	c.3105C>A	rs918241456		CN29	T	D	D	22.2	1	113,586	0.000009

SIFT is based on version 5.2.2; PolyPhen is based on version 2.2.2 release 405c; MutationTaster2021; CADD score is based upon Ensembl Ch37

^a^R118 also carries a rare variant in *KCNAB1* that is predicted to be deleterious as previously reported in Ibrahim et al. (2020) [39], although not listed here as no other sample in this cohort carries a rare, potentially deleterious variant in this gene

^b^R150 also carries a rare variant in *GABRG1* that is predicted to be deleterious as previously reported in Ibrahim et al. (2020), not listed here as no other sample in this cohort carries a rare, potentially deleterious variant in this gene

*GnomAD NFE* allele frequency of non-Finnish Europeans, *GnomAD* allele count is based upon v2.1.1 (exomes)

The genes carrying most variants across the cohort were *SNCAIP* with four variants found across 10 participants including p.Glu709Gln in five participants and p.Arg806His in three participants; *SCN9A* with six variants identified across seven participants, including p.Asn1256Ser identified in three participants and one participant carrying two potentially damaging variants p.Leu1278Val and p.Ile228Met; *ATP7B* with six variants across six participants; *CHAT* with three variants identified in six participants, including p.Arg561Gln in three participants; *KIF17* with three variants across four participants, including p.Arg111Gly in two participants; *SCN8A* with the p.Arg1026Cys variant present in four participants; *SYT9* with two variants across four participants, including p.Leu353Val identified in three participants; *CACNA1A* with one variant, p.Ala454Thr identified in three participants; and *SCN10A* with four variants across four participants. Related family members were available for segregation analysis in a subset of cases (Tables S3 and S4). However, the small pedigrees sizes, and the requirement for head trauma exposure to classify an individual as affected, limited the ability to draw any strong conclusions regarding variant segregation.

Fifteen variants identified in the concussion cohort were further genotyped in a migraine case–control cohort of 280 migraine cases and 280 age and sex-matched controls via an Agena MassARRAY plex to confirm their frequency in a population collected from a similar geographical background, or with some symptomatic overlap (i.e. migraine). As results presented in Table 4 show, the channel variants identified in the concussion cohort were confirmed to be rare in general populations, and they do not appear to be particularly associated with migraine which is a common symptom of concussion.

**Table 4:** Genotypic analysis of ion channel variants identified in concussion study in general migraine case–control cohort

Gene	Variant	gnomAD MAF (NFE)	No. variant alleles in migraine cases (*n*=280)/Frequency*	No. variant alleles in controls (*n*=280)/Frequency*
*ATP7B*	p.Asn41Ser	0.000486	0	0
	p.Val519Met	0.001123	1/0.00179	0
	p.Leu795Phe	0.000035	0	0
	p.Thr991Met	0.002359	2/0.00357	3/0.00536
	p.Val1234Phe	0	0	0
	p.Gly1287Ser	0.000009	0	0
*CACNB2*	p.Cys168Tyr	0.000018	0	0
	p.Leu453Phe	0.000422	1/0.00179	1/0.00179
*SCN8A*	p.Arg1026Cys	0.014730	2/0.00357	7/0.0125
*SCN9A*	p.Thr370Met	0.000010	0	0
	p.Asn1256Ser	0.007506	1/0.00179	0
	p.Leu1278Val	0.002073	1/0.00179	0
*SCN10A*	p.Arg844Cys	0.000018	0	0
	p.Val1287Ile	0.001110	0	0
	p.Ala1529Thr	0.000009	0	0

*If number (no.) of variant alleles is >0

Although all the variants we identified were predicted to be deleterious, there is insufficient evidence to suggest that any are pathogenic in a monogenic autosomal dominant manner, as the participant’s symptoms of concussion were precipitated with head trauma. However, the results suggest that variants across ion channels and neurotransmitter genes that may be sub-clinical could plausibly confer increased susceptibility to concussion and exacerbate post injury ionic fluxes post-TBI under limited energy/recovery conditions.

### Rare variant burden analysis on ion channel genes

Rare variant burden analysis focusing on the variants identified in the 16 ion channel genes from the Tier 1 and 2 analyses (Tables 1 and 2) was conducted to test whether there was enrichment (increased prevalence) of the damaging variants identified in the ion channel gene set in the concussion participants compared to a general population control group obtained from the gnomAD database (NFE) which most closely reflects the ancestry of the concussion cohort. As detailed in Table S5, in our concussion cohort we identified 43 different rare, amino acid changing variants in 16 ion channel and ion transporter genes, according to our filtering criteria. The overall prevalence of damaging alleles was rare (< 0.02) in both the case and control groups. In the concussion group, a total of 51 predicted damaging alleles at these loci were observed. This prevalence (0.64%) was ~5-fold greater than the prevalence of these alleles observed in the gnomAD population control group (0.12%) and significantly different by Fisher’s Exact test (Odds Ratio = 5.44, 95% CI [4.13,7.18], *P* < 0.0001). Gene-based burden testing was not conducted due to the limited size of the concussion cohort. Replication of these findings in independent, ancestrally matched case–control cohorts will be important for confirming the observed enrichment.

## Discussion

As disruption of ion homeostasis and synaptic neurotransmission represents a key feature of the neurometabolic response to TBI, we used a targeted, pathway-based approach to identify rare variants with potential functional significance in genes involved in these processes. Using Whole Exome Sequencing data from individuals with a history of concussion, we investigated whether rare variation in migraine-associated, ion channel, transporter, and neurotransmission-related genes may contribute to an increased vulnerability to concussion. 63% of participants who had sustained concussion carried at least one rare, potentially deleterious (based on in silico prediction) missense heterozygous variant in an ion channel or neurotransmitter-related gene. Furthermore, recurrent rare variants were observed in several prioritised genes across unrelated cases, with eight genes harbouring variants in four or more participants. Collectively, these findings highlight several genes with plausible involvement in concussion susceptibility and support further investigation of the functional consequences of the identified variants.

### Familial hemiplegic migraine genes

Causal genes for FHM – *CACNA1A* (FHM1), *ATP1A2* (FHM2), or *SCN1A* (FHM3) – were specifically included in the analysis due to the symptomatic overlap of these disorders with the neurologic symptoms that often present after concussion and the dysregulation of ion fluxes and neurotransmission that occurs in both migraine and concussion patients. We had previously found that a high proportion (35%) of hemiplegic migraine cases with pathogenic *ATP1A2* missense variants were noted to have incidence of concussion or concussion-related symptoms after mild head trauma [45]. These individuals had been excluded from the current study, and no further rare *ATP1A2* variants were identified in this cohort.

FHM patients with *CACNA1A* mutations may also be highly sensitive to head impacts [34, 35]. Three unrelated participants had a rare missense variant (p.Ala454Thr) in *CACNA1A*, which encodes the pore-forming subunit of the voltage-gated calcium channel Ca_V_2.1 [46] and plays an important function in controlling neurotransmitter release at presynaptic terminals [47–49]. p.Ala454Thr is annotated as likely benign in ClinVar, although it was previously found in a heterogeneous ataxic disorder [50], and shown to confer loss-of-function (LoF) effects by altering the voltage dependence of steady-state inactivation and interactions between Cav2.1 and SNARE proteins which led to decreased channel coupling to exocytosis [51]. Such functional effects could potentially contribute to dysregulation of the ion fluxes that occur in concussion.

A p.Arg1917Gly variant in *SCN1A*, which encodes the voltage-gated sodium channel Na_V_1.1, was identified in two unrelated participants. Notably, both participants also have a second rare missense variant in other genes of interest, i.e. *CHAT* (C2) or *CACNB2* (CN11). Although *SCN1A* p.Arg1917Gly is classified as likely benign in ClinVar, participant CN11 reported multiple concussions with ongoing photo- and phono-sensitivity and migraine with aura (age of onset 30 years). We therefore suggest that while this heterozygous variant may not lead to the severe FHM phenotype under normal conditions, head trauma may act as a precipitant of disturbance leading to the migraine with aura phenotype post-concussion, with or without additional variant burden in other ion channel or neurotransmission related genes.

### Ion channel genes and ion transporter genes

Expanded analyses of a panel of 353 ion channel and related genes identified potential causal variants in other ion channel genes in the concussion cohort. Rare variants were detected in genes encoding calcium channel α1 (*CACNA1B, CACNA1C, CACNA1D, CACNA1E, CACNA1I*) and auxiliary subunits (*CACNB2*), sodium channel α1 subunits (*SCN1A, SCN8A, SCN9A, SCN10A*), ATPases (*ATP7B*, *ATP10A, ATP13A4*), and potassium channel subunits (*KCNJ10, KCNT2*). The highest number of damaging rare variants in ion channel-related genes was detected in *SCN9A* (i.e. six different variants in eight cases).

#### Calcium channel genes

Cellular calcium influx is a key component of the neurometabolic cascade following a deceleration injury as highlighted by Giza and Hovda [24]. “*CACNA1x*” are a family of conserved genes that code the 10 different pore‐forming α1 subunits of voltage‐gated calcium channels (VGCCs). Including *CACNA1A* discussed previously, 18 rare variants were identified across seven VGCCs, with three participants carrying two missense variants in two different calcium channel genes (BBB12 – *CACNA1C* p.Asn2174Ser and *CACNA1E* p.Arg1932Trp; P186 – *CACNA1I* p.Asp2005Tyr and *CACNA1D* p.Asn1254Ser; and P200 – *CACNA1E* p.Ser1180Leu and *CACNB2* p.Cys169Tyr).

Three rare variants were identified in *CACNA1I*. We previously reported the p.Arg111Gly variant in R211, who had severe migraine and ataxia following head injury and a family history of migraine [39]. Functional testing of Ca_V_3.3 channels with the p.Arg111Gly variant by patch-clamp assay found that it reduced current density in comparison with wild-type [52]. The other two *CACNA1I* variants identified in unrelated cases should be considered for similar functional follow-up. The *CACNA1B* p.Arg1389His variant found in BBB19 has previously been linked to dystonia, albeit with conflicting results [53, 54]. Functional analysis of this variant in a study of a family with myoclonus-dystonia syndrome with cardiac arrhythmias showed that mutant channels carried less current when open compared with wild-type Ca_V_2.2 channels, consistent with a gain-of-function (GoF) [53].

Three variants each were found in nine different participants in the *CACNA1C, CACNA1D,* and *CACNA1E* genes, which encode the α1 subunits of Ca_V_1.2, Ca_V_1.3, and Ca_V_2.3 channels, respectively, all with important roles in a variety of neuronal processes [55]. *CACNA1C* has been associated with a spectrum of cardiac and neurological disorders including Timothy Syndrome [56]. The *CACNA1C* p.Asn2174Ser variant (annotated as p.Asn2091Ser in some transcripts) has been previously identified in a case of sudden unexplained death in the young (SUDY) and shown to exhibit minor kinetic alterations in functional tests [57]. *CACNA1D*, expressed in post-synaptic neurons, is associated with increased risk of neuropsychiatric disease and epilepsy [58], while *CACNA1E* Ca_V_2.3 channels initiate rapid synaptic transmission through high-voltage-activated, rapidly inactivating R-type calcium currents in the central nervous system (CNS), with de novo missense *CACNA1E* variants causal for developmental and epileptic encephalopathies [59].

#### Sodium channel genes

Sodium channels are involved in a variety of neurological disorders including migraine, epilepsy, and pain disorders. Furthermore, increasing evidence suggests that concussion can induce forms of sodium channelopathy [29, 60]. The detection of multiple variants in this class of genes in individuals that had sustained concussion may indicate that vulnerability to concussion, or more severe reactions post-concussion, could arise from functional variants which affect regulation of sodium ion fluxes.

*SCN9A* encodes the voltage-gated sodium channel subunit Na_V_1.7 which plays a significant role in nociception signalling and pain mechanisms. Pathogenic GoF variants in *SCN9A* that affect neuronal excitability have been associated with varying clinical presentations, including primary erythromelalgia, paroxysmal extreme pain disorders, and peripheral neuropathy [61–63]. *SCN9A* variants have also been associated with various epilepsy and seizure disorders [64, 65]. Six rare missense mutations at residues highly conserved across mammalian orthologues were identified in the *SCN9A* gene across the concussion cohort, four of which have been previously associated with known pain-related disorders [63, 66]. *SCN9A* p.Ile228Met has been functionally shown to impair slow-inactivation (i.e. GoF) and can increase the excitability of trigeminal and dorsal root ganglion neurons [66, 67]. Estacion and colleagues identified the variant in the three patients showing phenotypic diversity of pain disorders – one primary erythromelalgia, and two with small fibre neuropathy [66]. Participant CN10 with the p.Ile228Met variant reported a single concussion with ~18 months recovery time, however, suffered ongoing mental fatigue, visual disturbances, and occasional memory disturbances. Interestingly, they also have an *SCN9A* p.Leu1278Val variant. *SCN9A* variants including p.Leu1278Val have been found in Dravet syndrome patients alongside *SCN1A* mutations; Singh et al. hypothesised that carriers of *SCN9A* variants might either be asymptomatic or only present infrequently with febrile seizures due to incomplete penetrance and variable expressivity, however, may function as genetic modifiers in individuals with *SCN1A*-related Dravet syndrome, contributing to differences in disease severity and clinical presentation [64]. The *SCN9A* p.Asn1256Ser variant was found in three different participants reporting confusional migraine or migraine-like episodes following head injury.

The *SCN8A* p.Arg1026Cys variant was present in four unrelated participants, an allele frequency of 2.2% in the concussion cohort, while at ~1.4% in general population databases. *SCN8A* encodes the voltage-gated sodium channel subunit Na_V_1.6, and pathogenic variants can be classed as either LoF or GoF with variable consequences. Pathogenic GoF variants generally present with early-infantile epilepsies, while LoF variants generally lead to later-onset epilepsies, absence epilepsy, neurodevelopmental delays, and behavioural features such as attentional dysfunction [68, 69]. Interestingly, a swine model that had undergone experimental concussion showed widespread loss of Na_V_1.6 channel isoforms in white matter axons at 6 h and 72 h after injury, which recovered by two weeks post-injury, and may underlie the brain network dysfunction that occurs after concussion [29]. A loss of Na_V_1.6 and changes in nodes of Ranvier structures were similarly observed in the white matter of post-mortem human brain samples with TBI [29].

Four different rare *SCN10A* missense variants in four concussion participants were identified. *SCN10A* encodes the neuronal voltage-gated sodium channel subunit of Na_V_1.8, an important regulator of neuronal excitability. *SCN10A* pathogenic variants are most commonly associated with cardiac disorders [70], but also have been associated with cases of autism spectrum disorder [71, 72], pain perception and seizures [73], and small fibre neuropathy [63].

Some of the sodium channel variants we detected have previously been shown to have clinical relevance and/or functional effects. We hypothesise that the variants may contribute to relatively minor effects on sodium channel function, which, in the event of trauma, are sufficient to interfere with recovery of normal neuronal function and homeostasis. The finding of multiple rare and potentially damaging variants in sodium channel genes in our concussion cohort aligns with the proposal that sodium channelopathy underlies concussion symptoms [29, 60]. Our results could be particularly relevant in cases of multiple concussions to the finding that traumatic axonal injury from concussion induces a form of sodium channelopathy on axons that greatly exaggerates the pathophysiologic response to subsequent mild injuries [60].

#### ATPase genes

Three ATPase-related genes with multiple rare variants were identified in the study: *ATP10A* (*n* = 2 variants), *ATP13A4* (*n* = 1 variant, also present in an affected family member, Table S4), and *ATP7B* (*n* = 6 variants). The *ATP10A* p.Ala881Val variant in participant R170 who presented with confusional migraine following minor head injury had been previously reported [39], and now a second rare, likely damaging *ATP10A* variant (p.Glu81Gly) in a case presenting with migraine without aura and memory problems after several concussions was identified.

Pathogenic LoF variants in *ATP7B* cause the autosomal recessive Wilson disease due to impaired copper metabolism in which the basal ganglia and liver undergo changes that express themselves in neurological manifestations, and signs of cirrhosis, respectively [74]. Neurological manifestations include changes in personality and behaviour, similar to what can occur following TBI. We previously reported the heterozygous *ATP7B* p.Leu795Phe variant in patient R211 that presented with severe migraine and ataxia following head injury [39]. Included with the current study, we have identified six participants out of 93 who have had concussion with a heterozygous novel or rare *ATP7B* missense variants previously described as pathogenic, or of uncertain significance, for Wilson disease, while the frequency of heterozygous pathogenic *ATP7B* variants carriers in the population is estimated as only ~1 per 90 [75]. It is possible that heterozygous variants in this gene contribute to concussion susceptibility, with head trauma acting as trigger that unmasks the effects of the risk allele [39]. This is consistent with the established role of metal ions in neurophysiology, and the dysregulation in metal ion homeostasis that may occur following TBI [76].

#### Potassium channel genes

Potassium channels are a diverse ion channel group with five types defined by their activators: voltage-gated, calcium-activated, inwardly rectifying, ATP-sensitive and sodium-activated [77]. Rare and likely deleterious variants were identified in potassium channel genes *KCNJ10* and *KNCT2*. *KCNJ10*, a member of the inward rectifier-type potassium channel family (Kir4.1), has a role in maintaining the ionic environment in the extracellular space, acting as a modulator of neuronal activity and the glutamatergic system. Kir4.1 dysfunction has been linked to neurological conditions including depression and epilepsy [78], and it is downregulated in murine models of neurodegeneration, with resulting increased neuronal hyperexcitability a possible driver of neuronal loss [79]. We previously reported the p.Arg18Trp variant in R117 [39], who presented with recurrent episodes of pallor and vomiting following minor head injury, and sensitivity to loud noises and light for three weeks. CN32, with a *KCNJ10* p.Lys354Arg variant, had sustained multiple concussions, but no ongoing or long-term symptoms.

*KCNT2* encodes a sodium-activated potassium channel (also known as SLICK) which is widely expressed across the CNS, modulating membrane hyperpolarisation and adapting neuronal firing patterns [80]. Pathogenic *KCNT2* variants have been identified in development and epileptic encephalopathies (DEE) [80, 81], although no functional or clinical data is available for the p.Lys929Glu and p.Ile542Leu variants identified here.

Interestingly, another exploratory exome-based study of 50 concussion patients found multiple rare variants with predicted high impact in *KCNN3*, which encodes a calcium-activated potassium channel (K_Ca_2.3) that regulates neuronal excitability by dampening calcium mediated neuronal firing frequency [82]. It is possible that the functional effects of the channel variants identified in these studies are not significantly different from those of wild-type channels, except perhaps in the context of significant ionic dysregulation as occurs in concussion. Further functional assays would be required to test this hypothesis.

### Neurotransmission-related genes

As research has shown that following concussion there is a release of neurotransmitters such as glutamate [23], and that damaged neurons demonstrate reduced or inhibited neuronal signalling in some neuron subtypes [83], we hypothesised that genes in pathways regulating neurotransmission may also harbour genetic variants that play a role in concussion.

Rare variants were identified in various neurotransmission-related genes including: *CHAT* (p.Arg323His, p.Leu458Phe, p.Arg561Gln), *DAGLB* (p.Ile596Thr), *KIF17* (p.Glu807Ala, p.Arg111Gly, p.Glu65Lys), *NRG1* (p.Thr416Ser), *SNCAIP* (p.Val213Met, p.Arg621Cys, p.Glu709Gln, p.Arg608His), *SV2C* (p.Val321Glu, p.Asp456Asn), *SYT9* (p.Arg85Gln, p.Leu353Val) and *UNC13B* (p.Arg687Gln, p.Ser760Ile, p.Asn1035Lys). Further information on these genes is detailed in Supplementary Information 6. Several of these genes have been linked to cognitive function and particularly have been associated with Alzheimer’s and/or Parkinson’s disease. For example, ten participants in the concussion cohort had variants in the *SNCAIP* gene (five with p.Glu709Gln and three with p.Arg806His). *SNCAIP* encodes synuclein alpha-interacting protein or synphilin-1. It is enriched at synaptic terminals, has a role in regulating energy homoeostasis and cellular ATP levels, and is associated with alpha-synuclein and inclusion bodies in Parkinson’s disease [84, 85]. The relationship between Parkinson’s disease and head injury is not clearly understood, although some studies have shown increased risk of Parkinson’s disease in those who have suffered mild to moderate head injury. For example, a significant increased risk for Parkinson’s disease diagnosis later in life was found in a cohort of 47,483 individuals with a concussion (Odds ratio = 1.57, 95% CI [1.41,1.75], *p* < 0.001) [86]. A similarly large study of veterans also identified 56% increased risk of Parkinson’s disease in military veterans who had suffered mild traumatic brain injury [87]. These data add to the body of evidence suggesting that there may be some overlap of the genetic risk factors for concussion and Parkinson’s disease.

## Limitations

We acknowledge the relatively small size of the cohort (*n*=93), which means we have limited analyses to an overall neuronal vulnerability hypothesis rather than grouping concussion outcomes and post-concussion symptoms. Therefore, some findings may be related to specific outcomes rather than initial vulnerability to concussion. The cohort was heterogeneous, combining participants recruited via multiple pathways, including neurologist referred patients, military veterans, athletes, and self-reported cases. While these groups may differ in injury mechanism, concussion severity and symptoms, or other environmental exposures, the main aim of this exploratory study was to investigate rare variants in ion channel and neurotransmission-related genes in people who have sustained concussion, independent of the context in which the concussion occurred. This work will need to be extended and validated in larger TBI/concussion cohorts, and optimally with subgroup analyses performed with more clinically homogeneous concussion outcomes or sources of trauma.

Population allele frequencies for comparison with our concussion cohort were obtained from the gnomAD NFE reference dataset, which was selected as it was considered the most appropriate comparison given the predominantly European ancestry of the study participants, to reduce the potential for population stratification, and because it is one of the largest publicly available population resources for rare variant frequency estimation. Nevertheless, differences may arise through sample ascertainment, sequencing methodologies, and variant-calling pipelines. While sequencing of ancestrally matched controls on the same platform would be ideal, to minimise such influences, checks of all variants of interest included in the burden analysis were performed in IGV to ensure that the calls were good quality and reflected genuine variants.

Rather than a formal association study, our analytical approach was designed primarily for variant discovery and prioritisation. As numerous genes and variants were examined, the possibility of false-positive findings due to multiple testing should be considered. To reduce this risk, analyses were restricted to genes within biological pathways that have a well-established mechanistic role in concussion pathophysiology, and variants meeting stringent filtering criteria, including rarity, predicted functional impact, and recurrence in multiple unrelated participants. Nevertheless, replication in independent cohorts remains necessary. The limited sample size also meant we could not use a genome-wide approach for analysis or burden testing and acknowledge that targeting gene sets may miss important gene variants, or identify rare variants that are not necessarily linked to our hypothesis. Furthermore, not all individuals in the cohort had coding variants in the genes that were targeted for analysis, and our study does not preclude the role of other genes, non-coding or common variants, and non-genetic mechanisms in concussion.

## Conclusions

In summary, we have identified potentially deleterious coding variants in a series of neuronal ion channels and other synaptic genes in study participants who had sustained concussion/s. Many of these genes have been previously linked to disorders that show overlapping symptomology with post-concussion symptoms. We propose that while many of the identified heterozygote variants may be sub-clinical under normal conditions, dysfunction may be revealed after head trauma which disrupts ion homeostasis and requires recovery of ionic and excitatory/inhibitory balance leading to the symptoms commonly experienced post-concussion. Genes encoding ion channel proteins, especially those involved in calcium and sodium ion transport, are of particular interest. Importantly, several variants identified in the current study have previously been shown to alter ion channel or neuronal function in experimental systems, including variants in *CACNA1A*, *CACNA1B*, *CACNA1C, CACNA1I*, and *SCN9A*, supporting the biological relevance of the pathways identified. However, most variants reported here remain classified as candidate susceptibility variants, and their precise effects on neuronal excitability, ion homeostasis, neurotransmission, and concussion-related outcomes remain unknown. Candidate variants in genes involved in neurotransmission, and that have been associated with neurodegenerative disorders such as Alzheimer’s and Parkinson’s disease, were also identified in the study.

The most recent consensus statement of concussion in sport notes that while currently genetic testing may be a valuable research tool in the study of concussion, it is not yet suitable for routine use in clinical practice [3]. Accordingly, our results provide important genetic information for further investigation of the biological mechanisms underlying concussion susceptibility and outcomes. The identified genes and variants represent candidate factors for future validation studies and, following replication and functional characterisation, may contribute to the development of improved assessment of risk and prognosis, or therapeutic approaches for concussion.

## Supplementary Information

Below is the link to the electronic supplementary material.

Supplementary file1 (DOCX 147 KB)

Supplementary file2 (DOCX 43 KB)

Supplementary file3 (XLSX 16 KB)

Supplementary file4 (XLSX 20 KB)

Supplementary file5 (XLSX 19 KB)

Supplementary file6 (DOCX 19 KB)

## Data Availability

Data supporting the findings of this study are available within the paper and supplementary tables. Full participant sequencing data are not openly available due to ethics stipulations and reasons of privacy; however, specific information about genetic variants or genes in participants is available upon reasonable request to the corresponding author. Data are located in controlled access data storage at Queensland University of Technology. The novel sequence variant identified in this paper has been submitted to dbSNP for inclusion in the next available build (submission ID number 2137544578). When the build is completed by dbSNP, data will be available at https://www.ncbi.nlm.nih.gov/SNP/snp_viewTable.cgi?handle=GENOMICSRESEARCHCENT.
