# Supplementary material for: Investigating genetic susceptibility to concussion through rare variants in ion channel and neurotransmission genes
Source: J Neurol. 2026 Sep 12;273(10):591. doi: 10.1007/s00415-026-14139-8 (PMC13570918; doi:10.1007/s00415-026-14139-8)
Supplement: Supplementary file 1 [file 415_2026_14139_MOESM1_ESM.docx]

Investigating genetic susceptibility to concussion through rare variants in ion channel and

neurotransmission genes

Bridget H Maher, Neven Maksemous, Heidi G Sutherland, Robert A Smith, Omar Dabash, Annette Greenhow,

Fatima A Nasralla, Dale R Nyholt, Arn M J M van den Maagdenberg, Rod A Lea, and Lyn R Griffiths*

Genomics Research Centre, Centre for Genomics and Personalised Health, School of Biomedical Sciences,

Faculty of Health, Queensland University of Technology, Brisbane, Queensland, Australia

[*lyn.griffiths@qut.edu.au](mailto:*lyn.griffiths@qut.edu.au)

**Figure S1.** **Variant filtering workflow showing the number of variants retained after each filtering step.** Filtering via Ion Reporter^TM^ software (v5.12) included gene-set restriction via application of gene filter chains, protein-altering variant selection, rarity filtering (gnomAD non-Finnish European (NFE) minor allele frequency (MAF) < 0.02), pathogenicity prediction (using SIFT, Polyphen, MutationTaster, and CADD), Integrated Genome Viewer (IGV) confirmation, recurrent-gene filtering, and final prioritisation with respect to likely biological relevance. The final dataset consisted of 62 rare potentially deleterious variants across 24 genes identified in 59 participants.
