# Supplementary material for: Investigating genetic susceptibility to concussion through rare variants in ion channel and neurotransmission genes
Source: J Neurol. 2026 Sep 12;273(10):591. doi: 10.1007/s00415-026-14139-8 (PMC13570918; doi:10.1007/s00415-026-14139-8)
Supplement: Supplementary file 2 [file 415_2026_14139_MOESM2_ESM.docx]

Investigating genetic susceptibility to concussion through rare variants in ion channel and

neurotransmission genes

Bridget H Maher, Neven Maksemous, Heidi G Sutherland, Robert A Smith, Omar Dabash, Annette Greenhow,

Fatima A Nasralla, Dale R Nyholt, Arn M J M van den Maagdenberg, Rod A Lea, and Lyn R Griffiths*

Genomics Research Centre, Centre for Genomics and Personalised Health, School of Biomedical Sciences,

Faculty of Health, Queensland University of Technology, Brisbane, Queensland, Australia

[*lyn.griffiths@qut.edu.au](mailto:*lyn.griffiths@qut.edu.au)

***Supplementary Information 6. Extended discussion of neurotransmission-related genes with variants identified in the concussion cohort***

*CHAT* encodes the enzyme choline acetyltransferase (ChAT), which catalyses the synthesis of the neurotransmitter acetylcholine from acetyl coenzyme A and choline in neurons. Reduced ChAT activity has been associated with a number of disorders including Alzheimer’s disease and amyotrophic lateral sclerosis, and pathogenic variants in *CHAT* cause congenital neuromuscular disorders [1]. Of relevance to the current study, cholinergic dysfunction has been linked to cognitive outcomes from mTBI [2,3].

The *KIF17* gene belongs to the kinesin superfamily proteins (KIFs), which are molecular motors that use ATP to transport cargo along microtubules and primarily mediates fast anterograde transport. KIF17 selectively transports glutamate ionotropic receptor NMDA type subunit 2B (GRIN2B; NR2B) in vesicles along microtubules from the cell body to the dendrites of neurons, which are critical for synaptic plasticity and memory [4].

2-AG synthase diacylglycerol lipase β (*DAGLB*) encodes a key enzyme in the biosynthesis of endocannabinoid, 2-arachidonoylglycerol (2-AG) and downstream metabolic product arachidonic acid. In traumatic brain injury arachidonic acid derived mediators have been shown to drive inflammatory conditions [5] and recently multiple homozygous loss-of-function mutations in *DAGLB* have been linked to an early onset form of autosomal recessive Parkinsonism [6].

Two novel variants in *SV2C* were found in the concussion cohort. SV2C is an important regulator of dopamine release and is consistently altered in Parkinson’s disease [7]. Pathogenic variants in *SV2C* have been linked to Parkinson’s disease, particularly in Asian populations [8]. *SV2C* gene encodes synaptic vesicle glycoprotein 2C which is predicted to be active in synaptic function and involved in chemical synaptic transmission and neurotransmitter transport.

*UNC13B* encodes a presynaptic protein that promotes the priming of synaptic vesicles by acting through syntaxin. The protein also contains domains that are linked to calcium-triggered synaptic transmission [9], a feature of the neurometabolic cascade that follows concussion.

*NRG1* encodes the growth factor Neuregulin-1, and there are several proposed neuroprotective effects of *NRG1* after brain trauma. In the CNS, NRG1/ErbB4 signaling affects dendritic spine maturation and the formation of inhibitory synapses. NRG1/ErbB4 signaling also dynamically modulates GABAergic transmission via activation of neuronal nitric oxide synthase [10]. NRG1 has been shown to be upregulated in the days following injury [11], and several studies suggest a plausible role for NRG1 in recovery from concussion [12].

The *SYT9* gene is widely expressed in the brain and has been recently shown to act as a Ca^2+^ sensor that regulates the exocytosis of Substance P containing vesicles from striatal neurons [13]. The release of Substance P and binding to NK1 receptors have been demonstrated to mediate a variety of downstream processes post-concussion of traumatic brain injury [14].

**References**

1. Cai Y, Cronin CN, Engel AG, Ohno K, Hersh LB, Rodgers DW (2004) Choline acetyltransferase structure reveals distribution of mutations that cause motor disorders. EMBO J 23 (10):2047-2058. https://doi:10.1038/sj.emboj.7600221

2. Salmond CH, Chatfield DA, Menon DK, Pickard JD, Sahakian BJ (2005) Cognitive sequelae of head injury: involvement of basal forebrain and associated structures. Brain 128 (Pt 1):189-200. https://doi:10.1093/brain/awh352

3. Koshy Cherian A, Tronson NC, Parikh V, Kucinski A, Blakely RD, Sarter M (2019) Repetitive mild concussion in subjects with a vulnerable cholinergic system: Lasting cholinergic-attentional impairments in CHT+/- mice. Behav Neurosci 133 (4):448-459. https://doi:10.1037/bne0000310

4. Yin X, Feng X, Takei Y, Hirokawa N (2012) Regulation of NMDA receptor transport: a KIF17-cargo binding/releasing underlies synaptic plasticity and memory in vivo. J Neurosci 32 (16):5486-5499. https://doi:10.1523/JNEUROSCI.0718-12.2012

5. Harrison JL, Rowe RK, Lifshitz J (2016) Lipid mediators of inflammation in neurological injury: shifting the balance toward resolution. Neural Regen Res 11 (1):77-78. https://doi:10.4103/1673-5374.175046

6. Liu Z, Yang N, Dong J, Tian W, Chang L, Ma J, Guo J, Tan J, Dong A, He K, Zhou J, Cinar R, Wu J, Salinas AG, Sun L, Kumar M, Sullivan BT, Oldham BB, Pitz V, Makarious MB, Ding J, Kung J, Xie C, Hawes SL, Wang L, Wang T, Chan P, Zhang Z, Le W, Chen S, Lovinger DM, Blauwendraat C, Singleton AB, Cui G, Li Y, Cai H, Tang B (2022) Deficiency in endocannabinoid synthase DAGLB contributes to early onset Parkinsonism and murine nigral dopaminergic neuron dysfunction. Nat Commun 13 (1):3490. https://doi:10.1038/s41467-022-31168-9

7. Dunn AR, Stout KA, Ozawa M, Lohr KM, Hoffman CA, Bernstein AI, Li Y, Wang M, Sgobio C, Sastry N, Cai H, Caudle WM, Miller GW (2017) Synaptic vesicle glycoprotein 2C (SV2C) modulates dopamine release and is disrupted in Parkinson disease. Proc Natl Acad Sci U S A 114 (11):E2253-E2262. https://doi:10.1073/pnas.1616892114

8. Foo JN, Chew EGY, Chung SJ, Peng R, Blauwendraat C, Nalls MA, Mok KY, Satake W, Toda T, Chao Y, Tan LCS, Tandiono M, Lian MM, Ng EY, Prakash KM, Au WL, Meah WY, Mok SQ, Annuar AA, Chan AYY, Chen L, Chen Y, Jeon BS, Jiang L, Lim JL, Lin JJ, Liu C, Mao C, Mok V, Pei Z, Shang HF, Shi CH, Song K, Tan AH, Wu YR, Xu YM, Xu R, Yan Y, Yang J, Zhang B, Koh WP, Lim SY, Khor CC, Liu J, Tan EK (2020) Identification of Risk Loci for Parkinson Disease in Asians and Comparison of Risk Between Asians and Europeans: A Genome-Wide Association Study. JAMA Neurol 77 (6):746-754. https://doi:10.1001/jamaneurol.2020.0428

9. Michelassi F, Liu H, Hu Z, Dittman JS (2017) A C1-C2 Module in Munc13 Inhibits Calcium-Dependent Neurotransmitter Release. Neuron 95 (3):577-590 e575. https://doi:10.1016/j.neuron.2017.07.015

10. Wan C, Xia Y, Yan J, Lin W, Yao L, Zhang M, Gaisler-Salomon I, Mei L, Yin DM, Chen Y (2024) nNOS in Erbb4-positive neurons regulates GABAergic transmission in mouse hippocampus. Cell Death Dis 15 (2):167. https://doi:10.1038/s41419-024-06557-1

11. Stahl TJ, Palli R, Gill JM, Saionz EL, Bogner-Flatz V, Cheetham-West A, Merchant-Borna K, Pham DL, Strike L, Wright MJ, Thompson P, Bazarian JJ (2022) ErbB Signaling Pathway Genes Are Differentially Expressed in Monozygotic Twins Discordant for Sports-Related Concussion. Twin Res Hum Genet 25 (2):77-84. https://doi:10.1017/thg.2022.15

12. Kataria H, Alizadeh A, Karimi-Abdolrezaee S (2019) Neuregulin-1/ErbB network: An emerging modulator of nervous system injury and repair. Prog Neurobiol 180:101643. https://doi:10.1016/j.pneurobio.2019.101643

13. Seibert MJ, Evans CS, Stanley KS, Wu Z, Chapman ER (2023) Synaptotagmin 9 Modulates Spontaneous Neurotransmitter Release in Striatal Neurons by Regulating Substance P Secretion. J Neurosci 43 (9):1475-1491. https://doi:10.1523/JNEUROSCI.1857-22.2023

14. Vink RaC, F. (2023) The Role of Substance P and NK1 Receptors in Mild to Severe Traumatic Brain Injury: From CTE to ICP. Receptors 2 (4): 220-231
