# Supplementary material for: Investigating genetic susceptibility to concussion through rare variants in ion channel and neurotransmission genes
Source: J Neurol. 2026 Sep 12;273(10):591. doi: 10.1007/s00415-026-14139-8 (PMC13570918; doi:10.1007/s00415-026-14139-8)
Supplement: Supplementary file 3 [file 415_2026_14139_MOESM6_ESM.docx]

Investigating genetic susceptibility to concussion through rare variants in ion channel and

neurotransmission genes

Bridget H Maher, Neven Maksemous, Heidi G Sutherland, Robert A Smith, Omar Dabash, Annette Greenhow,

Fatima A Nasralla, Dale R Nyholt, Arn M J M van den Maagdenberg, Rod A Lea, and Lyn R Griffiths*

Genomics Research Centre, Centre for Genomics and Personalised Health, School of Biomedical Sciences,

Faculty of Health, Queensland University of Technology, Brisbane, Queensland, Australia

[*lyn.griffiths@qut.edu.au](mailto:*lyn.griffiths@qut.edu.au)

**Supplementary Table 3. Available family members of index concussion participants**

| **Index participant ID** | **Relative ID** | **Relationship** | **Concussion history of relative** |
| --- | --- | --- | --- |
| CN1 | CN2 | Sibling of CN1 | Multiple concussions throughout sporting life from 15-26 years, varied response times, multiple weeks on one occasion. |
|  | CN3 | Parent of CN1 | Single concussion at age 40, 2-4 week recovery time. |
| CN7 | CN8 | Sibling of CN7 | Response to minor Head Trauma: migraine/prolonged headaches/visual distrubances/memory problems, brother (CN7) suffered same response. |
|  | CN35 | Parent of CN7 | Concussion as a result of car accident. No ongoing symptoms. |
|  | CN36 | Parent of CN7 | Concussion as a result of car accident. Ongoing symptoms include Headache and Visual Aura. |
| CN15 | CN12 | Sibling of CN15 | No concussions. |
|  | CN37 | Cousin of CN15 | Had a single concussion, not diagnosed by physician no ongoing symptoms, recovery time unsure. |
|  | CN38 | Cousin of CN15 | 2 concussions, 2 week recovery time no ongoing symptoms. |
| CN18 | CN21 | Parent of CN18 | Had a single concussion, not diagnosed by physician no ongoing symptoms, recovery time 10 mins. |
|  | CN20 | Parent of CN18 | No concussions. |
|  | CN17 | Sibling of CN18 | No concussions. |

**Supplementary Table 4. Segregation of rare ion channel or neurotransmitter related gene variants**

| **Variants** | **Family ID** | | | |
| --- | --- | --- | --- | --- |
|  | CN1 (A) | CN2 (A) | CN3 (A) |  |
| *SCN10A* p.Val1287Ile | + | - | - |  |
| *SYT9* p.Leu353Val | + | + | + |  |
|  |  |  |  |  |
|  | CN7 (A) | CN08 (A) | CN35 (A) | CN36 (A) |
| *CACNA1A* p.Ala454Thr | + | + | + | - |
| *KCNT2* p.Lys929Glu | + | - | - | - |
|  |  |  |  |  |
|  | CN15 (A) | CN12 (UA) | CN37 (A?) | CN38 (A) |
| *CACNA1D* p.Gly2034Ser | + | - | - | - |
|  |  |  |  |  |
|  | CN18 (A) | CN21 (A?) | CN20 (UA) | CN17 (UA) |
| *SCN8A* p.Arg1026Cys | + | + | - | + |
| *ATP13A4* p.Asn978His | + | + | - | - |

Concussion status: A = affected; UA = unaffected; A? = Possible concussion
